# A Public Health Response against *Strongyloides stercoralis*: Time to Look at Soil-Transmitted Helminthiasis in Full

**DOI:** 10.1371/journal.pntd.0002165

**Published:** 2013-05-09

**Authors:** Alejandro J. Krolewiecki, Patrick Lammie, Julie Jacobson, Albis-Francesco Gabrielli, Bruno Levecke, Eugenia Socias, Luis M. Arias, Nicanor Sosa, David Abraham, Ruben Cimino, Adriana Echazú, Favio Crudo, Jozef Vercruysse, Marco Albonico

**Affiliations:** 1 Instituto de Investigaciones en Enfermedades Tropicales - Universidad Nacional de Salta, Oran, Argentina; 2 Instituto de Patología Experimental/CONICET, Salta, Argentina; 3 Division of Parasitic Diseases and Malaria, Centers for Disease Control and Prevention, Atlanta, Georgia, United States of America; 4 Bill & Melinda Gates Foundation, Seattle, Washington, United States of America; 5 Department of Control of Neglected Tropical Diseases, World Health Organization, Geneva, Switzerland; 6 Department of Virology, Parasitology and Immunology, Faculty of Veterinary Medicine, Ghent University, Merelbeke, Belgium; 7 Fundación Mundo Sano, Buenos Aires, Argentina; 8 Ministerio de Salud Pública de la Provincia de Salta, Salta, Argentina; 9 Department of Microbiology and Immunology, Kimmel Cancer Center, Thomas Jefferson University, Philadelphia, Pennsylvania, United States of America; 10 Subsecretaría de Salud, Municipalidad de Zárate, Zárate, Argentina; 11 Centre for Tropical Diseases, Sacro Cuore Hospital, Negrar, Italy; 12 Ivo de Carneri Foundation (IdCF), Milan, Italy; Swiss Tropical and Public Health Institute, Switzerland

## Abstract

*Strongyloides stercoralis* infections have a worldwide distribution with a global burden in terms of prevalence and morbidity that is largely ignored. A public health response against soil-transmitted helminth (STH) infections should broaden the strategy to include *S. stercoralis* and overcome the epidemiological, diagnostic, and therapeutic challenges that this parasite poses in comparison to *Ascaris lumbricoides*, *Trichuris trichiura*, and hookworms. The relatively poor sensitivity of single stool evaluations, which is further lowered when quantitative techniques aimed at detecting eggs are used, also complicates morbidity evaluations and adequate drug efficacy measurements, since *S. stercoralis* is eliminated in stools in a larval stage. Specific stool techniques for the detection of larvae of *S. stercoralis*, like Baermann's and Koga's agar plate, despite superiority over direct techniques are still suboptimal. New serologies using recombinant antigens and molecular-based techniques offer new hopes in those areas. The use of ivermectin rather than benzimidazoles for its treatment and the need to have curative regimens rather than lowering the parasite burden are also unique for *S. stercoralis* in comparison to the other STH due to its life cycle, which allows reproduction and amplification of the worm burden within the human host. The potential impact on STH of the benzimidazoles/ivermectin combinations, already used for control/elimination of lymphatic filariasis, should be further evaluated in public health settings. While waiting for more effective single-dose drug regimens and new sensitive diagnostics, the evidence and the tools already available warrant the planning of a common platform for STH and *S. stercoralis* control.

## Introduction

Soil transmitted helminthiasis (STH) affects up to one in four individuals in the world, disproportionately affecting impoverished populations without access to adequate water, sanitation, and opportunities for socioeconomic development [1]. Efforts to control the impact of STH are based on public health interventions that have periodic anthelminthic treatment, primarily of children, as the foundation for school or community based interventions. Attention has historically focused on just four species of STH, *A. lumbricoides*, *T. trichiura*, and hookworms (*Ancylostoma duodenale* and *Necator americanus*). Due to the challenge of measuring the disease burden and monitoring the control intervention, the role of *S. stercoralis*, which is as much an STH as the other four by its standard definition, has been neglected in the repertoire of strategies to reduce the burden of these neglected tropical diseases (NTDs) through public health interventions [2]. Main characteristics of STH and *S. stercoralis* infections are illustrated in Table 1.

**Table 1:** Principal features of STH and *S. stercoralis*.

Feature	Major STH				*S. stercoralis*
	*A. lumbricoides*	*T. trichiura*	*A. duodenale*	*N. americanus*	
Multiplication within the host/autoinfection	−	−	−	−	+++
Morbidity acute/chronic	+/+ ++	+/+++	++/++++	+/+++	++/++++
Potential fatality	+	−	++	+	++++
Main diagnostic stage	Egg	Egg	Egg	Egg	Larvae
Therapeutic goal	Cure or decreasing worm load	Cure or decreasing worm load	Cure or decreasing worm load	Cure or decreasing worm load	Cure
Outcome measurement	Cure rate and egg reduction rate	Cure rate and egg reduction rate	Cure rate and egg reduction rate	Cure rate and egg reduction rate	Cure rate

The aim of the present review is to discuss why *S. stercoralis* has been overlooked in the management of STH through large-scale administration of anthelminthic drugs (preventive chemotherapy), and to highlight the aspects of this helminthic infection that justify its inclusion in a more comprehensive approach to STH control. Among the reasons for the traditional approach, which excludes/ignores *S. stercoralis*, are the non-standard approaches needed to diagnose *S. stercoralis*, the different drugs and treatment regimens needed, and the paucity of data on subtle and chronic morbidity that sustain the lack of clear goals for an intervention targeting the infection [3]–[5]. The description of the difficulties described in the following sections of this article should however be viewed as a challenge to overcome rather than a justification for the maintenance of the status quo. This challenge will most likely find success through the current strategy of aggregating and combining treatments and integrated control rather than developing single disease interventions when feasible [6].

## Clinical Importance of *S. stercoralis*

Strongylodiasis is best known in the developed world for the severe consequences of the hyperinfection syndromes linked to immunosuppression caused by diseases like lymphomas, leukemias, or the use of corticosteroids [7]. This clinical entity, which in resource-poor countries is probably associated with widespread malnutrition, is probably just the tip of an iceberg of unknown size. Defining the denominator of patients infected by *S. stercoralis* and the assessment of associated morbidity including the proportion of those that suffer hyperinfection in a given community is essential to better identify risk factors, understand pathogenesis, and plan control measures in its natural setting.

As noted by other authors, the disability-adjusted life year (DALY) system represents a poor estimate of the true burden and morbidity of STH overall [8]. For *S. stercoralis*, this is further complicated by the challenges of clinical follow-up, as its infection does not trigger anemia nor does it have any clinical marker that can be easily diagnosed and monitored. Attempts to demonstrate the subtle morbidity associated with *S. stercoralis* infections are also limited by the difficulties in correctly identifying uninfected control groups with the poorly sensitive direct diagnostic tools such as stool examinations that are the current standard. A recent report was however able to identify through questionnaires and stool evaluations that individuals infected by *S. stercoralis* were more likely to complain of stomach ache [9]. Pathology evaluations of the gastrointestinal tract indicate that there is a lack of lesions in the mucosa in most infections, which only becomes apparent in severe infections. These changes include an inflammatory response that ranges from congestive catarrhal enteritis to an edematous and ulcerative enteritis, reflecting the wide spectrum of symptoms found in endemic areas, with a significant proportion of individuals without acute intestinal complaints [10], . Non specific urticaria, a bothersome symptom, is a very frequent finding (up to 100% in a report of 52 cases), but is rarely reported spontaneously by the infected individuals [12]. More information on the impact on the quality of life impaired by these infections, particularly in endemic populations rather than just in travelers [9], should be the basis for the control of strongyloidiasis with the goal of reducing the related morbidity, and not just as a means of lowering the chances of developing hyperinfection. In summary, without good reliable data on infection, morbidity, and indisputable causal links between them, it has been challenging to make the public health argument for the response against *S. stercoralis*.

## Diagnostic Challenges

Most of the difficulties posed in the measurement of *S. stercoralis* infections and its consequences rely on the challenging aspects of its diagnosis, which ultimately affect its incorporation into a control package with the other STH. In terms of diagnostics, Kato Katz (the diagnostic method recommended by WHO) and McMaster are techniques made to detect (and quantitate) eggs, which do not detect *S. stercoralis* larvae [13]. Even new developments in this area, like the FLOTAC, an improvement of the McMaster, focus on egg detection and fail to detect the presence of *S. stercoralis*, which is diagnosed in stool exams through the identification of its larval stages [14], [15]. At present the two most appropriate diagnostic tools for the diagnosis of *S. stercoralis* are the Baermann and the Harada Mori methods, although their sensitivity is not optimal; the agar plate method is more sensitive but also more expensive and laborious [3].

There are also important therapeutic implications in terms of clinical trial design and evidence-based recommendations that emerge from the challenges of diagnosing *S. stercoralis*. Starting with the inclusion criteria for such trials, categorizing a patient as positive for *S. stercoralis* is a lot easier than categorizing that same patient as negative or cured using either one, two, or three stool exams as test of cure, as has been very carefully demonstrated in a well controlled population studied with eight stool exams [16]. Such issues should question the interpretation and conclusions of every clinical efficacy trial that uses these stool techniques as test of cure. These challenges stress the need for a new generation of diagnostics. The incorporation of real-time PCR assay for multiple STH, including *S. stercoralis*, could be an improvement to overcome this obstacle due to its reported high sensitivity, although this warrants further validation [17].

The complexities in diagnosis have led to the challenge of demonstrating the burden of disease of this parasite and this may explain why *S. stercoralis* is still neglected from the public health perspective. Innovative approaches for the diagnosis of STH aim at solving the weaknesses of traditional methods that depend on stool collection; such weaknesses are not only linked to the low sensitivity and specificity of the different techniques but also to the difficulties in getting several stool samples per patient for analysis. While it is clear that, especially for *S. stercoralis*, several samples increase sensitivity [16], large scale programs have accepted the use of a single sample, given the complexity and costs of collecting and processing multiple samples. This results in significant underestimations of the true prevalence of infection while not completely eliminating the difficulties of collecting stools.

*S. stercoralis* serologic assays can simplify the diagnosis of this infection and overcome the poor sensitivity of single stool exams, both for diagnosis of individual patients and also for defining infection prevalence at the community level [18]. The introduction of assays based on recombinant antigens that can be produced in large quantities offers attractive alternatives to the use of crude antigen, which requires the maintenance of laboratory animals with chronic infections or stool collections from infected individuals for antigen production. Recent field evaluations with a 31-kDa recombinant antigen (termed NIE), which has no cross reactivity with other STH, have shown improved sensitivity compared to a variety of stool evaluations in a single stool exam [19]. A commercial ELISA assay with recombinant antigen could be the ideal product for *S. stercoralis* diagnosis, as other methods either need the cumbersome collection of crude antigen, or like the immunofluorescence antibody test (IFAT) are too dependent on the operator skills and on the performing laboratory, and are thus difficult to standardize and replicate on a large scale. It is still unknown whether antibody levels measured with any of these assays correlate with worm burden. The possibility of using these assays to conduct sero-surveys is currently limited to *S. stercoralis* due to the lack of antigens for *A. lumbricoides*, *T. trichiura*, or hookworms with similar performance, and calls for further research in the search of antigens for the other STH. The available diagnostics test for STH and *S. stercoralis* are illustrated in Table 2.

**Table 2:** Diagnostic techniques for the diagnosis and quantification of STH.

Type of Method	Technique	*S. stercoralis*	STH		
			*A. lumbricoides*	Hookworms	*T. trichiura*
Parasitological methods	Direct exam	+	+	+	+
	Sedimentation concentration	++	++	++	++
	Baermann (+/− charcoal culture)	+++	−	+^a^	−
	Agar plate	++	−	+^a^	−
	Harada Mori	++	−	+^a^	−
	McMaster	—	+++^b^	+++^b^	+++^b^
	Kato Katz	—	+++^b^	+++^b^	+++^b^
	FLOTAC	—	+++^b^	+++^b^	+++^b^
Serology	Crude antigen ELISA	+++	−	−	−
	IFAT	+++	−	−	−
	Recombinant antigens (LIPS, ELISA)	+++	−	−	−
PCR		++	++	++	++

a For larvae detection and species identification.

b Quantitative techniques.

IFAT, indirect immunofluorescence antibody test; LIPS, luciferase immunoprecipitation systems.

## Treatment Challenges

Current therapy is another issue to be revised if *S. stercoralis* is to be considered in the spectrum of targeted parasites amenable to public health control. Neither of the recommended drugs for use in large scale interventions to control STH infections, which include the benzimidazoles albendazole and mebendazole, levamisole, and pyrantel/oxantel have any significant activity against *S. stercoralis*, at least in single doses as recommended for preventive chemotherapy interventions [1], [20]. In terms of treatment goals, while lowering parasite burden in the group of individuals with moderate and high worm burdens is, from an arguable public health point of view, a reasonable goal for *A. lumbricoides*, *T. trichiura*, and hookworms [21], [22], this is not true for *S. stercoralis*. The peculiar life cycle of *S. stercoralis*, and specifically this worm's unique (among STH) ability to reproduce within the human host, makes anything but parasitologic cure a treatment failure and therefore, any measure of parasite load reduction would not be a measure of success as for the other STH [23]. This last reason is what makes a drug like albendazole, with cure rates of approximately 40% when used in single-dose regimens, an unsatisfactory option for *S. stercoralis* [24], [25]. This difference in life cycles results in having at a maximum one adult worm per each invasive egg or larva that infects the host for *A. lumbricoides*, *T. trichiura*, and hookworms, but infinite numbers (resembling the situation in protozoan and bacterial infections) for *S. stercoralis*. Larvae from this parasite hatch in the stool, rapidly evolve into infective L3 filariform larvae, and re-infect the same individual, perpetuating the infection in healthy hosts and through the expansion of this re-infection step, overwhelm the host in the context of immune suppression [7]. In reference to immune protection, it is clear that acquired immunity develops in humans to infection with *S. stercoralis*, on the basis of the antibody responses that develop to the infection [26]. Furthermore, acquired protective immunity to the infection has been described extensively in animal models [27]. However, the lifelong nature of infections in humans argues that host immunity may limit worm burden, but it is not sufficient to eradicate it.

Ivermectin, the current standard of care for the treatment of *S. stercoralis* infections, showed superiority against thiabendazole in terms of safety and similar efficacy [28], [29]. Due to its widespread use in lymphatic filariasis and onchocerciasis control programs, ivermectin has a well determined safety profile. It is however restricted in pediatric populations, limiting its use according to different criteria of age, weight, and/or height (older than either 3 or 5 years old, taller than 90 cm, or above 15 kg of weight) depending on the country. The reasons for this are related to potential toxicity in the central nervous system seen most commonly but not only in Collie dogs, which is however related to a genetic predisposition of those dog species and has not been observed in humans [30]. The exposure of breastfed infants during the treatment of their mothers (ivermectin is contraindicated in pregnancy and the first week post partum [13]), does not add significant information about exposure in these children despite the significant passage through milk (40% of the plasma levels), since this amount has been calculated to be just 2 to 4 µg/kg for the average infant—a dose 50 to 100 times less than the usual dose [31]. Emerging data suggest that the use of this drug could be a powerful tool to prevent rheumatic fever through the treatment of scabies [32], a reasonable option for head lice, and a component of a combination regimen with mebendazole or albendazole for the treatment of *T. trichiura* [33]. Thus ivermectin is turning into an essential drug in the pediatric pharmacopeia. Hence, demonstrating the safety of this drug in the group of 3 to 5 year olds to realize its full therapeutic possibilities is a reasonable and important step. Despite the widespread use of ivermectin in many countries endemic for lymphatic filariasis, onchocerciasis, and STH, unfortunately very few studies have looked at the impact of those interventions on strongyloidiasis in areas where this infection is co-endemic [34].

Initiatives like the one by WHO proposing multicentric, properly controlled, powered, and monitored clinical trials employing medicines from a known and reputable drug manufacturer are a necessary initial step to acquire definitive data about all the anthelminthics included in the essential drugs list (albendazole, mebendazole, levamisole, and pyrantel) in order to produce biologic and statistically sound evidence. This process has already been used to generate data on albendazole [22], is ongoing with mebendazole, and is in the pipeline for pyrantel/oxantel. Similar initiatives with ivermectin in developed countries against *S. stercoralis* in settings where infection can be properly controlled and reinfection can be prevented, are needed as starting points to produce solid evidence. A multicentric randomized trial in a clinical setting to assess efficacy and safety of different therapeutic regimens of ivermectin (single dose of 200 µg/kg versus 200 µg/kg for 2 consecutive days, repeated 14 days after the first dose) is ongoing (ClinicalTrials.gov Identifier: NCT01570504) and will give valuable answers to clinical and public health questions. In addition, this clinical trial will answer the question on the most sensitive and specific serological diagnostic assay and its value as marker of cure. Therapeutic efficacies with different drug regimens and combination for STH and *S. stercoralis* infections are illustrated in Table 3.

**Table 3:** Cure rates and egg reduction rates (mean %) of anthelminthics recommended by WHO, administered in single dose against STHs.

Drug	Dose	Rate	*S. stercoralis* a	*A. lumbricoides*	Hookworms	*T. trichiura*	Refs
Albendazole	400 mg	CR	—	88–98.4	78.4–100	10–52.7	[22], [24], [33], [46]
	—	ERR	—	86.5–100	64.2–100	40.3–50.8	—
Mebendazole	500 mg	CR	—	95–96.5	22.9	19–36	[24], [33]
	—	ERR	—	—	—	66.7–92.8	—
Ivermectin	200 µg/kg	CR	56.6–68.1	78.4–94.2	—	35.1–44.3	[28], [46], [47]
	—	ERR	—	94.3–100	—	42.7–86.8	—
Pyrantel	10 mg/kg	CR	—	88	31	28.1	[24]
	—	ERR	—	87.9	56.4–75	52	—
Levamisole	2.5 mg/kg or 80 mg	CR	—	91.5	10–38.2	9.6	[24]
	—	ERR	—	—	—	41.5	—
Albendazole/ivermectin	400 mg/200 µg/kg	CR	56.6–68.1^b^	78.1–100	78.4–100^b^	38–79.6	[33], [46], [47]
	—	ERR	—	99.5–100	100	68–97.5	—
Mebendazole/ivermectin	500 mg/200 µg/kg	CR	56.6–68.1^b^	96.5^b^	22.9^b^	55.196.7	[33]
	—	ERR	—	—	—	—	—

a Studies considered are only those that used antibody responses rather than parasitologic evaluations as test of cure.

b In view of the lack of control studies with these combinations, values refer to the efficacy observed with the administration of the most effective drug of the combination, used as monotherapy.

CR, cure rates; ERR, egg reduction rates.

## Control of *S. stercoralis*

Epidemiologic studies looking into the distribution of *S. stercoralis* in communities have shown prevalence peaks in adolescents, remaining stable in adults, with a similar distribution as hookworms. Some studies have shown no gender difference and others have found it more prevalent in males, possibly representing differential exposure [9], [35], [36]. Findings of higher burden in the adult population challenge the current policies of focusing interventions (and also drug donations) in school-age and preschool-age children. WHO guidelines offer a clear stepwise approach to the community based treatment of STH through anthelminthic therapy, with the 20% and 50% thresholds of combined prevalence for any of the major STH triggering the use of preventive chemotherapy interventions once or twice a year, respectively [13]. While this strategy is in use around the world and delivering measurable benefits, there is room for further study of this strategy in order to provide scientific support to the expansion or modification of this approach. Among these unsolved areas is the definition of an appropriate prevalence threshold that should trigger community treatments for *S. stercoralis*, considering the particularities of the life cycle and treatment goals discussed above. The search for new diagnostic tools for *S. stercoralis* should not hamper the development of strategies for the implementation of control programs. The use of the available, albeit moderately sensitive, direct diagnostic tools in sentinel sites should allow predicting a good enough picture of the distribution of *S. stercoralis* that could justify a therapeutic intervention. More evidence and data are needed, however, to define such prevalence thresholds.

Most published literature concerning this helminth (fewer than 150 articles) refers to clinical cases of hyperinfection, most of them from industrialized and middle-income countries. A crude estimate of this number with the quoted worldwide prevalence of 30 million to 100 million cases of strongyloidiasis means that for every case report there are approximately 200,000 to 700,000 cases, mainly in developing countries [37]. Another issue currently not contemplated in the recommendations for preventive chemotherapy programs against STH in humans, in contrast to what happens in veterinary medicine, is the timing of treatment based on climatic factors in order to prevent the emergence and proliferation of resistant clones through the maintenance of “wild type” clones in refugia [38]. Caution should however be used while trying to derive conclusions from clinical trials in the setting of public health interventions where causality and the weight of each component of the control programs must be considered. Controversies around meta-analyses aimed at answering key questions about STH management highlight the problems faced by trying to apply the evidence based medicine standards constructed mainly on the results and conclusions of randomized clinical trials, to complex interventions that are usually multiple and influenced by several factors, which sometimes are setting-specific and prevent the generalizability of the findings [39]. Translating results of properly powered and designed trials to define safety and efficacy into implementation programs, is dependent on the ability to measure the effectiveness of programs to facilitate proper decision making. The use of established primary care programs might offer opportunities to tailor universal recommendations by moving into trials and field interventions where multiple dosing regimens, pharmacovigilance, and monitoring activities could be implemented. An example of this model is ongoing in northwestern Argentina, where in the Department of Oran, Province of Salta, monitoring and evaluation of STH preventive chemotherapy interventions is based on a regimen containing albendazole and ivermectin. This program, combines stool analysis with a newly validated NIE-ELISA serologic assay (for *S. stercoralis*), and is being implemented by a primary care network with provincial coverage that through sanitary agents ensures at least four house visits annually. Community based interventions like this are allowing the potential integration of deworming and monitoring activities into the care of other NTDs (leishmaniasis, dengue, Chagas, and leprosy are prevalent in the area) and in a larger picture, public health care in all its aspects [36].

Overlapping endemicities, many with shared risk factors are the rule; new tools with the capacity to generate epidemiological and program impact data across a broader spectrum of target organisms are therefore needed. A multiplex bead-based technology that measures antibody responses to several agents in a single serum sample in a single well, is able to potentially produce up to 9,600 pieces of data from a single 96-well plate, with the possibility of evaluating a wide variety of health determinants simultaneously [40]. Such an assay might open up new options to program monitoring and evaluation efforts [41]. A similar approach through the use of molecular biology techniques measuring stool DNA in assays set for multiple enteric parasites (Multiplex) including *S. stercoralis*, *Entamoeba histolytica*, *Giardia intestinalis*, and *Cryptosporidium spp.* is also a promising tool for the evaluation of STH. This assay has an improved sensitivity in a single stool specimen, as shown by Basuni et al. in a real-time PCR assay for the simultaneous detection of *A. duodenale*, *N. americanus*, *A. lumbricoides*, and *S. stercoralis* [17]. PCR is a new diagnostic tool that still requires proper evaluation in the diagnosis of *S. stercoralis* infections, particularly in field studies, with its limitations and potential challenges. With reference to the intermittent shedding of *S. stercoralis* larvae in stools, it is still unclear whether this is true in absolute terms or if it is a phenomenon observed due to the fluctuating levels of larval output above and below the level of sensitivity of the different stool techniques, and may therefore, be overcome by this molecular-based sensitive technique. Multiplex molecular diagnostic devices measuring parasite DNA in stools can measure an array of intestinal parasites, and could give a full picture of the polyparasitism in endemic communities [42]. Such tools could be the new frontier of diagnostics that lay the foundation for innovative therapeutic options.

A great deal of progress has been made on development of vaccines against human infections with hookworms, beginning with animal studies and currently involving clinical trials [43]. Efforts to develop vaccines against human infection with *S. stercoralis* have been limited, although there have been excellent results in an animal model using a single recombinant diagnostic antigen as the vaccine against *S. stercoralis* [44]. The efficacy of this vaccine in humans has not been tested.

There is a variety of areas of research that need to provide the necessary answers to improve the actions taken against STH and identify the new implementation components that would make a comprehensive approach to STH and *S. stercoralis* control feasible. As an initial step, the identification of the most adequate diagnostic tools along with the safest and most effective treatment regimen to be implemented in a package with the other STH appears to be the most urgent need; this should be followed by the development and incorporation of new strategies and technology for the monitoring and evaluation. Drugs are at the center of such investigations: recent progress in the availability of mebendazole and albendazole through donation programs and the commitment of funds for their distribution pose a new emphasis in the need to identify the biomedical answers that will allow the best utilization of these newly available resources [45]. Ivermectin and albendazole, historically used for lymphatic filariasis control/elimination, already have a spectrum of activity against multiple parasites, including STH and *S. stercoralis*, intestinal protozoa, and ectoparasites, and the potential impact of this combination with different formulations and dosages should be further evaluated in public health settings.

**Five Key Papers in This Field**

- Olsen A, van Lieshout L, Marti H, Polderman T, Polman K, et al. (2009) Strongyloidiasis–the most neglected of the neglected tropical diseases? Trans R Soc Trop Med Hyg 103: 967–972.
- Lammie PJ, Moss DM, Brook Goodhew E, Hamlin K, Krolewiecki A, et al. (2012) Development of a new platform for neglected tropical disease surveillance. Int J Parasitol 42: 797–800.
- Dreyer G, Fernandes-Silva E, Alves S, Rocha A, Albuquerque R, et al. (1996) Patterns of detection of Strongyloides stercoralis in stool specimens: implications for diagnosis and clinical trials. J Clin Microbiol 34: 2569–2571.
- King CH (2010) Health metrics for helminthic infections. Adv Parasitol 73: 51–69.
- Ramanathan R, Burbelo PD, Groot S, Iadarola MJ, Neva FA, et al. (2008) A luciferase immunoprecipitation systems assay enhances the sensitivity and specificity of diagnosis of Strongyloides stercoralis infection. J Infect Dis 198: 444–451.

**Key Learning Points**

- Direct parasitological techniques for the diagnosis of *S. stercoralis* infections have suboptimal sensitivity, affecting adequate prevalence measurements, burden of disease estimations, and clinical trials design.
- The incorporation of *S. stercoralis* in the preventive chemotherapy strategy in place for other soil transmitted helminthiasis requires special adjustments in the drug regimens. Ivermectin is the drug of choice; albendazole and mebendazole have no significant activity in single drug regimens.
- The life cycle of *S. stercoralis*, which replicates within the human host, makes cure rather than lowering the worm burden, the appropriate therapeutic goal.
- Although multiplex molecular-based diagnostics and optimal treatment regimens for *S. stercoralis* and STH infections should be pursue as a pressing need, their development should not delay the planning and implementation of strategies to control strongyloidiasis and STH with the existing tools.
